# Plant cell wall characterization using scanning probe microscopy techniques

**DOI:** 10.1186/1754-6834-2-17

**Published:** 2009-08-24

**Authors:** John M Yarbrough, Michael E Himmel, Shi-You Ding

**Affiliations:** 1 Chemical and Biosciences Center, National Renewable Energy Laboratory, Golden, Colorado, USA

## Abstract

Lignocellulosic biomass is today considered a promising renewable resource for bioenergy production. A combined chemical and biological process is currently under consideration for the conversion of polysaccharides from plant cell wall materials, mainly cellulose and hemicelluloses, to simple sugars that can be fermented to biofuels. Native plant cellulose forms nanometer-scale microfibrils that are embedded in a polymeric network of hemicelluloses, pectins, and lignins; this explains, in part, the recalcitrance of biomass to deconstruction. The chemical and structural characteristics of these plant cell wall constituents remain largely unknown today. Scanning probe microscopy techniques, particularly atomic force microscopy and its application in characterizing plant cell wall structure, are reviewed here. We also further discuss future developments based on scanning probe microscopy techniques that combine linear and nonlinear optical techniques to characterize plant cell wall nanometer-scale structures, specifically apertureless near-field scanning optical microscopy and coherent anti-Stokes Raman scattering microscopy.

## Background

Lignocellulosic biofuels have been widely proposed as the most promising route for the sustainable and renewable production of liquid transportation fuels today [1]. There are millions of tons of lignocellulosic biomass, such as wood chips, agricultural residues, and grasses that could be converted to simple sugars and then fermented to transportation fuels. One key bottleneck for the current bioconversion technologies used to produce biofuels is the high cost and low yield of fermentable sugars (mainly glucose and xylose) derived from plant biomass. The cell walls in plant biomass are composed primarily of polysaccharides and a non-fermentable polyphenolic fraction, lignin. Cellulose is the major plant cell wall polysaccharide and consists entirely of non-branched β-1,4-linked glucose. The hemicelluloses comprise a variety of branched and esterified polysaccharides containing xylose, mannose, arabinose, galactose, and glucose linked by various glycoside bonds [2]. The dominant hemicellulose in grasses and hard woods, the main energy feedstocks, is arabinoxylan. Pectins are defined by the presence of uronic acids as major components [3]. Although lignin biosynthesis is less understood than the biosynthesis of polysaccharides in plant cell walls, it has been established that the composition of lignin in higher plants is derived from three monolignols: *p*-coumaryl, *p*-coniferyl, and *E*-sinapyl alcohol. These components are thought to play a vital role in the function and stability of the plant cell wall and constitute, in part, it's recalcitrant nature [2].

In concert, these biopolymers form the complex networks comprising the plant cell wall, where cellulose constitutes the core of the relatively rigid microfibrils and other cell wall polymers form 'matrixing' materials binding the system into a kind of 'liquid polymer crystal'. To deconstruct the plant cell wall and liberate these mixed sugars efficiently, the insoluble structural carbohydrates present must be first available to, and then converted to soluble sugars by cellulolytic and hemicellulolytic enzymes. In order to enhance enzyme digestibility, current biomass conversion technologies use thermaland/or chemical pretreatment to make the cell walls more amenable to these enzymes, although the detailed mechanisms of how these enzymes act on the complex plant cell wall substrate remains poorly understood.

As the primary structural component of the plant cell wall, cellulose forms the cores of plant cell wall microfibrils. The biophysical properties of cellulose are based almost entirely on how these glucan chains are packed (that is, cellulose can form the polymorphs Iα, Iβ, II, III and IV), which vary depending on the size of the fiber, the fiber length, the chain angle, and the solvent in which it is studied. Nevertheless, the structures of cellulose reported in the literature are primarily based on either highly modified or regenerated plant cellulose, or large cellulose crystals produced by algae or animals. The native structure of plant cell wall cellulose remains unknown primarily because insufficient tools are available to study cellulose microfibrils (2 to 10 nm in diameter) *in situ *or *in vivo *[4-6]. Even the number of cellulose chains in the elementary fibril is not known [7], although most previous work has proposed a 36-chain model based primarily on a single cellulose synthase complex or 'rosette' containing 36 subunit enzymes. There is evidence that cellulose microfibril formation is under dynamic cellular control regulated, at least in part, by the density and arrangement of the synthetic sites, as well as the identity and availability of other cell wall polymers that can co-aggregate with the forming cellulose fibrils [4]. Cellulose fibrils form an essential scaffold for many plant cell walls and their absence disrupts the hierarchical assembly of other cell wall components [8], such as hemicelluloses, pectins, and even lignins. Readers can find comprehensive recent reviews about plant cell wall synthesis elsewhere [3,6,9].

Plant cell wall structural elements are on the order of nanometers and, unfortunately, traditional optical microscopy does not allow direct observation of these materials. It is now clear that a better fundamental understanding of how these materials assemble to form the plant cell wall is critical. In order to accomplish this objective, characterization of these structures with regard to their chemical and physical features at the nanometer scale is necessary. Unfortunately, very few microscopy techniques permit direct measurement at this high spatial resolution without extensive and time-intensive sample preparation that can potentially damage or alter the plant material. Traditionally, these measurements have been made with scanning electron microscopy (SEM) and transmission electron microscopy (TEM). Sample preparation for TEM involves the embedding of the plant specimens into a resin for stabilization and then thin sectioning to a suitable thickness. For high-resolution SEM the sample must be made conductive, which is normally accomplished by coating biological specimens with vaporized metal or carbon. It is believed that these sample preparation procedures used for the plant materials can damage and ultimately change the native structure of the plant cell wall. With the recent introduction of high-pressure freezing and freeze substitution methods for preserving cells for ultrastructural analysis, electron microscopy has become a valuable tool for correlative imaging. However, there are still limitations with these imaging techniques and often the reduction of resolution is the resulting compromise [10-13].

To this end, scanning probe microscopy (SPM) provides a critical imaging modality that offers the ability to investigate these materials at the nanometer scale with essentially no sample preparation or perturbation, using a distinctive 'biophysical' methodology. In this context, we will review the principles of SPM and its application to imaging plant cell walls and cellulose crystals. Furthermore, we provide a discussion regarding the possible benefits of combing SPM with tip-enhanced near-field spectroscopy, as well as discussing potential correlations between SPM and traditional electron microscopy.

## Scanning probe microscopy and its application to imaging cellulose and the plant cell wall microfibril

SPM utilizes the ability to measure the interaction between a fine physical probe and the surface of the sample. Atomic force microscopy (AFM) and near-field scanning optical microscopy (NSOM) are two examples of SPM techniques that can obtain a spatial resolution at the sub-micron level (that is, between 0.1 nm and 100 nm). These techniques permit the direct characterization of the surface of biological systems with high spatial resolution and minimal sample preparation. They are ideal tools for characterizing the ultrastructure and biochemistry of the plant cell wall because many critical features of the cell wall lie within their detection range.

AFM is typically used to measure the interaction forces between the probe tip's radius of curvature and the sample surface. The tip radius is typically 5 to 10 nm, but can be as small as 1 nm. These tips are usually made from silicon or silicon nitride. AFM systems typically use an optical feedback circuit whereby a laser spot is reflected off the cantilever holding the tip and the deflection of the tip is measured as the tip is rastered across the sample surface. AFM can be used to measure primarily the physical or topographical, as well as some chemical, properties of the sample surface. NSOM; however, is typically used to measure the optical (and thus chemical) properties of materials in registry with their topography. This is accomplished by utilizing a tapered optical fiber as the probe. Since an optical fiber probe is used, NSOM has the ability to optically characterize (with wavelengths in the visible spectrum) a sample on the submicron level by permitting simultaneous injection of light into, or collection of light from the sample. This ability allows the optical properties of the sample, fluorescently tagged labels, and other optical techniques to be mapped in registry with topography at the nanometer scale.

All tip-based scanning probe microscopy uses an auxiliary gap-width sensing mechanism (for example, mechanical shear force, optical detection) in a feedback loop allowing a fixed tip-sample separation to be maintained while rastering the probe along the sample surface. This method allows scanning with small tip-sample separations without damaging the tip or the sample. Unfortunately, this type of scanning mechanism can give rise to artifacts, arising from cross talk between topographic and other signals caused by the gap-width control mechanism.

Along with the introduction of artifacts associated with a moving tip, there are other challenges associated with SPM. For example, as it is the surface of the material being probed, there is no direct measurement of the bulk properties of the material. Also, in most probe-type microscopy experiments there is a tendency for the probe tip to physically disturb molecules (especially biomolecules) that are not firmly attached to the substrate surface [14]. Finally, hysteresis plays a vital role in the repeatability of scanning probe microscopy, because it can lead to image distortions and drift of the sample [15]. With regard to both AFM and NSOM, steps can and should be taken to aid in determining whether or not tip artifacts influence the images. Considerable literature exists addressing this subject and it is not within the scope of this article to delve into greater detail about the techniques used for determining the presence of tip artifacts and strategies for overcoming these artifacts. Instead, the reader is directed to several articles that offer greater detail on this subject [16-26].

**Figure 1:**
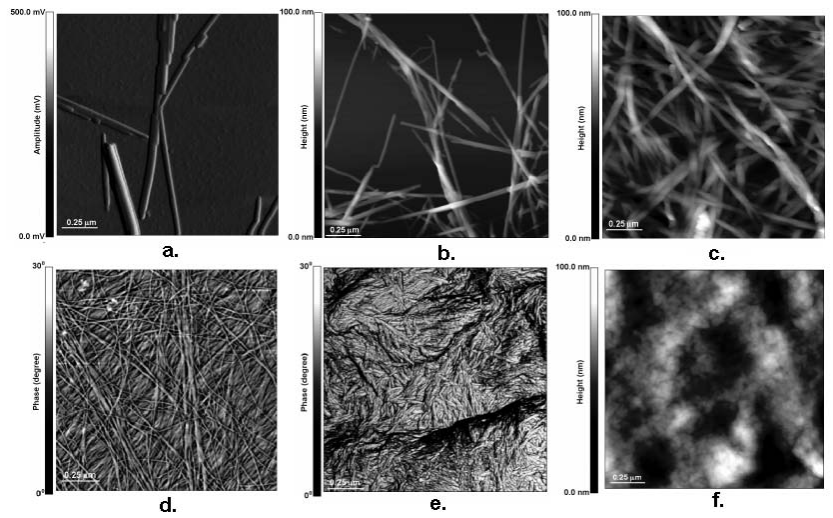
**Atomic force microscopy images of cellulose and plant cell wall microfibrils**. **a**. *Valonia*; **b**. tunicate; **c**. bacterial microcrystalline cellulose; **d**. maize parenchyma cell wall (reproduced from [28] with permission) **e**. Avicel; **f**. phosphoric acid treated cellulose (amorphous) (reproduced from [29] with permission).

Recently, there has been enhanced interest in using SPM, in particular AFM, to study the physical and chemical properties of biological materials, including plant cell walls. The literature reflects considerable work demonstrating the application of AFM to plant cell walls usually studied in both native and thermal chemically pretreated forms. Figure 1 demonstrates the practicality of using AFM to characterize the size and structure of cellulose preparations ranging from microcrystalline to amorphous forms (Figure 1). Shown in Figure 1a are examples of cellulose from *Valonia*, an alga that produces large, primarily Iα crystals that are approximately 20 nm in diameter. Figure 1b shows examples of cellulose from the tunicate, an animal, which are also large crystals, but these are primarily in the Iβ form. In contrast, the bacterium *Acetobacter xylinum *produces ribbon-like cellulose microfibrils (see Figure 1c). Figure 1d shows examples of the primary cell walls of higher plants (for example, maize), where cellulose takes the form of small microfibrils that are 3 to 5 nm in diameter [27]. In contrast, commercial cellulose preparations (for example, Avicel) are usually composed of microfibrils that appear as small crystals or bundles ranging from 100 to 200 nm in length, and 5 to 10 nm in diameter (Figure 1e). Under extreme conditions, cellulose can also be converted to a completely amorphous form when treated with concentrated phosphoric acid (Figure 1f) [29].

**Figure 2:**
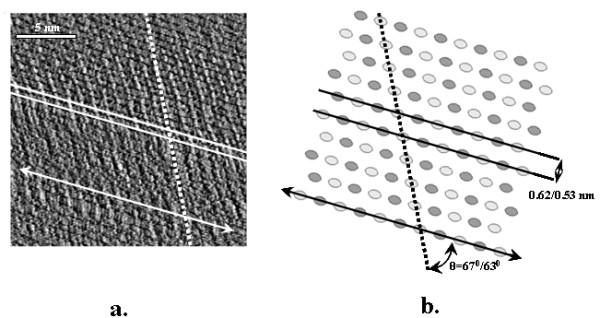
**Atomic force microscopy error image of Valonia cellulose surface show similar spacings with cellulose Iα**. **a**. Atomic force microscopy image taken in contact mode, **b**. A schematic drawing of the triclinic form of cellulose I. Reproduced from [30] with permission.

As previously stated, a significant amount of work has been reported using SPM to characterize and understand the complexity of cellulose. In 1997, Baker and co-workers [30] first reported an AFM study of cellulose microcrystals obtained from *Valonia*. Multiple measurements of the surfaces of the *Valonia *cellulose crystals were performed with a spatial resolution of approximate half a nanometer (Figure 2). Using 'deflection' mode imaging and 2D fast Fourier transforms of the data set of AFM images, it was demonstrated that there are three different regular spacings observed for the surfaces of *Valonia *cellulose Iα microcrystals. These spacings were interpreted as the glucose interval (0.52 nm), as well as cellobiose repeats (1.04 nm) and, less clearly, repeats apparently matching the inter-molecular chain spacing (0.6 nm). Although these apparent spacings seemed to agree with the deduced surface structure of cellulose Iα [30], they could also be related to known SPM tip artifacts. To eliminate potential artifacts, the cellulose surface was imaged in both propanol and water, and the scan direction was changed to avoid the typical scanning directional artifact. This work further suggested that these selected SPM artifact-identifying procedures were critical for verifying and strengthening the accuracy of the SPM data.

Kirby *et al*. [32] performed AFM measurements on several different plant cell wall materials that were taken from homogenized Cox orange pippin apples, water chestnuts, Bintji potato, and Amsterdamse bak carrot. Their work demonstrated the feasibility of using AFM to probe the molecular architecture of hydrated plant cell walls and also showed that sample preparation (in this case, freeze-thawing) could result in minor differences in the structure before and after treatment [32]. Their measurements showed clear evidence that the cell wall consisted of a laminated structure with different layers of fibers orientated in different directions, which supported the general assumption that typical plant cell walls are polylaminate structures. Kirby *et al*. also demonstrated that the isolated fibers found in the top layer were thicker than fibers that were ordered into aligned arrays or layers. This apparent thickness difference between fibers was attributed to a probe-broadening artifact [32].

Davis and Harris [33] performed AFM measurements on cell walls from onion and *Arabidopsis thaliana*. Their measurements showed tightly interwoven microfibrils, which in both species were approximately the same width. In addition to these measurements, they went on to determine microfibril diameters in samples that had been subjected to different treatment conditions. It was found that removal of pectic polysaccharides could improve the accuracy of measurements of the microfibril dimensions. These treatments consisted of using either a combination of *trans*-1,2-diamino-cyclohexane *N, N, N', N'*-tetraacetic acid (CDTA) with sodium carbonate or KOH. It was also reported from nuclear magnetic resonance (NMR) studies that the CDTA mixture did not affect the size or crystallinity of cellulose, whereas the KOH solution did alter the ordering of the cellulose and resulted in the production of both cellulose II and amorphous cellulose, along with possible swelling of the microfibrils. Different diameters of microfibrils were reported from different plant materials, which ranged 4.4 to 7.9 nm depending on the treatment used. Interestingly, these microfibrillar diameters were smaller than those reported previously (~25 nm) by Kirby *et al*. [31], the latter of which could be attributed to tip-broadening effects. Kirby and co-workers then suggested the use of height measurements to avoid the potential probe-broadening artifacts. Davis and Harris [33] also investigated the tip-broadening problem by performing multiple scans at different scales (magnifications). They were able to conclude that tip artifacts were unlikely contributors to the contours of the microfibrils observed, because they were not consistent throughout the image and they were seen in other scans of the same sample.

We recently reported [27,28] AFM imaging of the maize parenchyma cell wall surface. In this study, mature parenchyma cell walls from naturally senescent stem pith were imaged without any chemical treatment. The thick fibers that appeared to exist only in the uppermost surface of some parenchyma cell walls were also observed, which were consistent with the observations of Kirby *et al*. [32]. These structures were not found in cell walls in which the microfibrils appeared to be heavily coated with matrices suspected of containing hemicelluloses and pectins. We found that the larger fibers observed in the top layer of the cell wall appeared to be bundles of smaller fibers, which did not appear to result from tip-broadening as interpreted by Kirby *et al*. [32]. We maintained that they were bundles of newly synthesized cellulose elementary fibers (CEFs), which we later termed 'macrofibrils'. We also reported seeing similar random orientation of the microfibrils reported by Davis and Harris [33]. From these studies, we further reported the diameter of the CEF was 3 to 5 nm measured using AFM height imaging, which was smaller than that measured by previous AFM studies. This measured diameter seemed consistent with the general model of a 36-chain elementary fibril [27]. These models suggested that the elementary fibril is synthesized from one cell wall 'rosette' containing 36 cellulose synthase enzymes (CesA). As with the other AFM measurements reported here, we performed additional tests to eliminate the possibility of tip artifacts. These procedures included calibration of each tip with a reference gold particle sample, interchanging of tips, and a consistent scan-size strategy to ensure reproducibility.

AFM is a powerful tool that can provide high topographic resolution. However, one of the limitations of AFM is its poor chemical resolution capability, especially for imaging complex biomaterials, such as plant cell walls. As previously stated, these plant materials could be used for the production of lignocellulosic biofuels; however, the greatest barrier for commercial processes is overcoming the natural resistance of cell walls to deconstruction. To accomplish this task in a cost-effective manner, a better understanding of the fundamental chemical structure of the plant cell wall as well as the overall effects of pretreatment is needed.

Traditional microscopy and spectroscopy techniques can readily characterize these plant materials in bulk form (for example, at the macroscopic scale). Unfortunately, these diffraction-limited spectroscopic techniques are proving to be a barrier to advancing our fundamental understanding of these materials. It is now clear that in order to study the fundamental structure of cell walls at the nanometer scale, more advanced scanning probe techniques are needed. Ideally, such techniques would combine diverse spectroscopic techniques, including fluorescence, Raman scattering, and nonlinear optical processes, such as two-photon fluorescence, second harmonic generation, and third harmonic generation. With this objective in mind, recent developments in combining SPM techniques and linear or non-linear spectroscopy appear promising for achieving the required high spatial and chemical resolution, simultaneously.

## New developments based on the SPM technique and tip-enhanced near-field optical spectroscopy

High optical and/or chemical and spatial resolution can be achieved in NSOM using a tapered optical fiber with a sub-wavelength aperture. This sub-wavelength aperture (approximately 100 nm) is formed at the end of the tapered optical fiber by evaporating aluminum on all sides of the tapered sides of the optical fiber. There are several other methods to form this sub-wavelength aperture and each method has its advantages and disadvantages (for detailed methods, see [21,34]. However, smaller and larger apertures can be obtained and the size of the aperture typically governs the optical resolution, as well as the topographical resolution.

Optical microscopy is traditionally used to collect these spectroscopic signals in the far field, or to be more precise, above the diffraction limit of light where light begins to interfere with itself. As these systems operate in the far-field zone, they all have a spatial resolution of approximately λ/2 (that is, the Rayleigh criterion). This situation prohibits our ability to optically resolve and/or distinguish features smaller than the diffraction limit. This objective is a big challenge when optical microscopy is used to characterize plant cell wall constituents that are a few nanometers in size. Therefore, the signals collected by optical microscopy represent an ensemble average composed of signals from many different constituents in the plant cell wall. To increase spatial resolution, these optical techniques and SPM can be combined, which then permits measurements to be made below the diffraction barrier (within the near-field zone). These measurements could be performed on a sub-wavelength scale (that is, <<λ) where the spatial resolution is limited only by the size of the scanning probe. Recent achievements in understanding the interaction of light beyond the diffraction limit has led to an explosion of interest in using apertureless SPM to collect optical information beyond the diffraction limit of light [35].

The typical aperture on the end of an NSOM probe is approximately 100 nm in diameter; however, these probes can reach tip diameters as small as 20 nm. As these tips are made from an optical fiber the tip itself is very fragile and easily damaged, and this can lead to unwanted tip artifacts [34]. Along with the fragility of the NSOM probes, their collection efficiencies are very low, typically on the order of 10^-6^, which results in a requirement for long integration times to collect the optical signal. Due to these limitations of the optical probe used in NSOM, the apertureless probe with tip enhancement (TE) technique is more common today for the collection of optical signals below the diffraction limit of light. NSOM based on local field enhancement was first proposed in 1985, even before the invention of the atomic force microscope [35]. Today, this method involves using either a solid metal tip or a metalized AFM tip made by the electrochemical etching of a thin wire or by coating the tip with silver (usually a noble metal, preferably silver or gold) [36]. The optical signal generated can be strongly enhanced by this metal coating, which significantly improves the NSOM by drastically reducing the integration time and increasing the signal-to-noise ratio [37]. The detailed physics behind using TE-NSOM has been reviewed in elsewhere [35].

Considerable work is being done within this field today and these apertureless techniques are being used to perform most of the optical characterization within the near field. Fragola *et al*. [38] used apertureless near-field fluorescence microscopy on dye-doped polystyrene spheres immersed in liquid. The primary challenge they found was to distinguish the fluorescence signals from the far field (optical signal) of the polystyrene spheres and from the near field (optical component). They further used a lock-in amplifier that was synchronized to the tip oscillation frequency and demonstrated the ability to collect fluorescence in the near field only from the polystyrene spheres with high signal-to-noise ratio and high lateral resolution [38]. Furthermore, these measurements were performed in solution, which provided a promising approach for characterizing biological samples. For the case of plant cell walls, for example, in theory NSOM could be used in a similar way to characterize the compartmentalization and distribution of cell wall matrix polymers, as well as chemically distinct microfribrils arranged in layers.

## Tip-enhanced Raman spectroscopy

Surface-enhanced Raman scattering (SERS) has also been used to characterize organic molecules. Typically, SERS requires the deposition of a thin metallic layer onto the sample, which improves the Raman signal intensity by several orders of magnitude. This technique was developed because spontaneous Raman scattering suffers from a very low-scattering cross-section (typically, 1 in 10^6 ^photons account for the Raman signal) [36]. Therefore, deposition of a metallic layer (typically silver) depends critically on the substrate preparation, severely limits the applicability and renders quantitative measurement almost impossible. Another problematic factor is that the deposition of a thin layer of metal on a biological sample usually destroys it [37]. The surface-enhanced Raman techniques are thus unfavorable for characterizing biomolecules and perhaps plant cell walls, or at least must be employed with care.

Hayazawa *et al*. [37] first demonstrated tip-enhanced near-field Raman imaging using an apertureless metallic probe. Their experiment used aggregates of Rhodamine-6F and Crystal Violet molecules deposited onto a cover slip covered with an 8 mm-thick silver film. This silver film was used to enhance the surface Raman scattering effect, as well as to reduce the fluorescence background generated by energy transfer from the molecules to metal [36]. These measurements were performed in contact (tip in contact with the sample) and non-contact modes. The Raman scattering spectra were also collected from the focused spot. Interestingly, the Raman spectra collected in contact mode contained the inherent near-field Raman scattering induced by the metallic tip, which was absent in non-contact mode. These workers also reported that the integration times were 1 second with a low level of excitation (230 μW) for each organic molecule, which demonstrated the capability of TE-NSOM Raman imaging of organic molecules. Stöckle and co-workers [36] also used a sharp metal tip rastered over the sample with an AFM. They performed measurements on a thin Brilliant Cresyl Blue (BCB) layer and a C_60 _thin film that was drop-coated onto a glass substrate using both a metalized AFM probe and an electrochemically etched gold metal wire. In both experiments, they collected the Raman spectrum with contact and non-contact modes. The Raman signal increased more than 30 times when the metalized tips were brought into contact with these thin films with a spatial resolution on the order of 50 nm.

Along with employing these apertureless techniques to perform traditional optical spectroscopy, apertureless techniques are also being used in conjunction with non-linear optical techniques, such as two-photon excitation fluorescence (TPEF). TPEF spectroscopy utilizes two photons of energy to excite a fluorophore, resulting in the emission of a fluorescent photon at a higher energy than either of the two excitatory photons. The fluorescence yield scales as *E*^4 ^for TPEF, which is similar to the scaling for strong SERS [39]. Sánchez *et al*. [40] demonstrated the feasibility of collecting near-field TPEF using a metal tip on *J*-aggregates of pseudoisocyanine (PIC) dye molecule suspended in polyvinyl sulfate. A mode-locked Ti:Sapphire laser providing 100 fs pulses at 830 nm was used to demonstrate a spatial resolution ranging between 15 and 30 nm for these dyes. Near-field TPEF can also be obtained using metalized AFM probes. For example, Nieman *et al*. [38] used a gold-coated AFM probe on a dried coumarin I film mounted on a cover slip that had been degreased in acetone and methanol. A mode-locked Ti:Sapphire laser with a pulsed width of approximately 200 fs at a center wavelength of approximately 790 nm was used as the excitation source for this work. Nieman *et al*. [40] also reported short integration times, strong fluorescence yield, and both an optical and topographical spatial resolution of 43.8 nm, which is well below the diffraction limit. We conclude the TE-Raman AFM imaging may have sufficient resolution to chemically characterize plant cell wall compartments such as cellular scale features, but not microfibrils.

## Near-field coherent anti-Stokes Raman scattering microscopy

Another widely used non-linear optical technique is coherent anti-Stokes Raman scattering (CARS). CARS is a third-order non-linear four-wave mixing optical process involving two lasers, a pump laser at a frequency of ω_p _and a Stokes laser at a frequency of ω_s_. The resulting signal from these two lasers is at the anti-Stokes frequency of 2*ω*_*p *_- *ω*_*s *_which is generated in the phase-matching directions [41]. The CARS signal is achieved when these two lasers' beams are collinearly added and tightly focused onto a sample. This creates a composite electric field beating at the frequency *ω*_*p *_- *ω*_*s*_. If this beating frequency matches a specific vibrational Raman frequency within the molecules it causes this vibrational Raman frequency to oscillate, thereby producing a strong excitation across the whole focal volume [42]. Recently, there has been considerable interest in performing CARS on biological samples. The benefit of CARS is it can excite specific vibrational Raman modes within the biological system when the frequency difference (*ω*_*p *_- *ω*_*s*_) between the pump and the Stokes laser are tuned to the resonance of a specific Raman-active molecular vibration. Due to the coherent nature of the CARS possesses, CARS has higher sensitivity than spontaneous Raman microscopy.

Ichimura *et al*. [43] demonstrated the feasibility of performing near-field CARS using a metalized AFM tip on adenine molecules in a nanomeric DNA network structure, and tuned the pump and stokes frequencies to excite the 1337 cm^-1 ^Raman mode corresponding to the adenine (ring-breathing mode of diazole). These workers observed a background CARS signal, which they attributed to the third-order non-linear susceptibility of silver (for example, due to local four-wave mixing). Noble metals used as the probe can also pose another problem besides local four-wave mixing; they can also generate a white light continuum which is induced by multi-photon excited photoluminescence due to the recombination radiation between electrons near the Fermi level and photo-excited holes in the d band [43]. Unfortunately, the combination of these background signals competes with the CARS process. The CARS signal in this experiment largely surpassed the background signal and so the authors were able to collect a strong CARS signal. They reported that they had imaged both topographically and optically the DNA network with fast integration times (ranging between 3 to 12 minutes) and a spatial resolution of about 250 nm. The same workers [43] also performed near-field CARS on single-wall carbon nanotubes (SWNT) using a gold-metalized silicon AFM tip. Kawata *et al*. [44] further improved the spatial resolution to about 150 nm; in this case, the pump and stokes frequencies were tuned to excite the Raman mode corresponding to the G-band of the SWNTs at 1581 cm^-1^.

**Figure 3:**
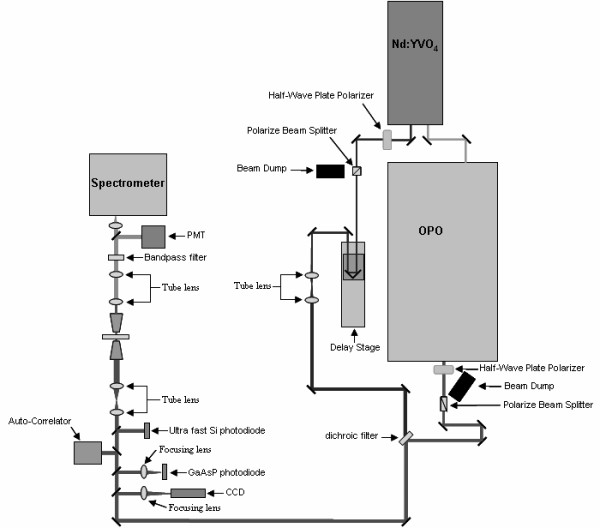
**Schematic diagram showing the details of the CARS setup that was used to chemically characterize the components of the plant cell wall**.

**Figure 4:**
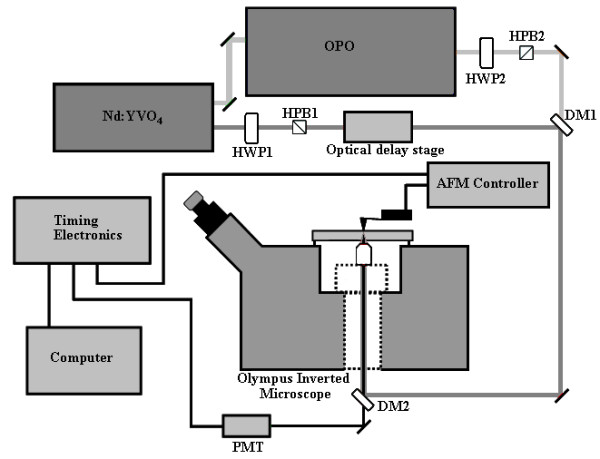
**Schematic showing the basic coupling of the existing CARS setup with a Veeco BioScope II AFM mounted to an Olympus Inverted microscope**.

**Figure 5:**
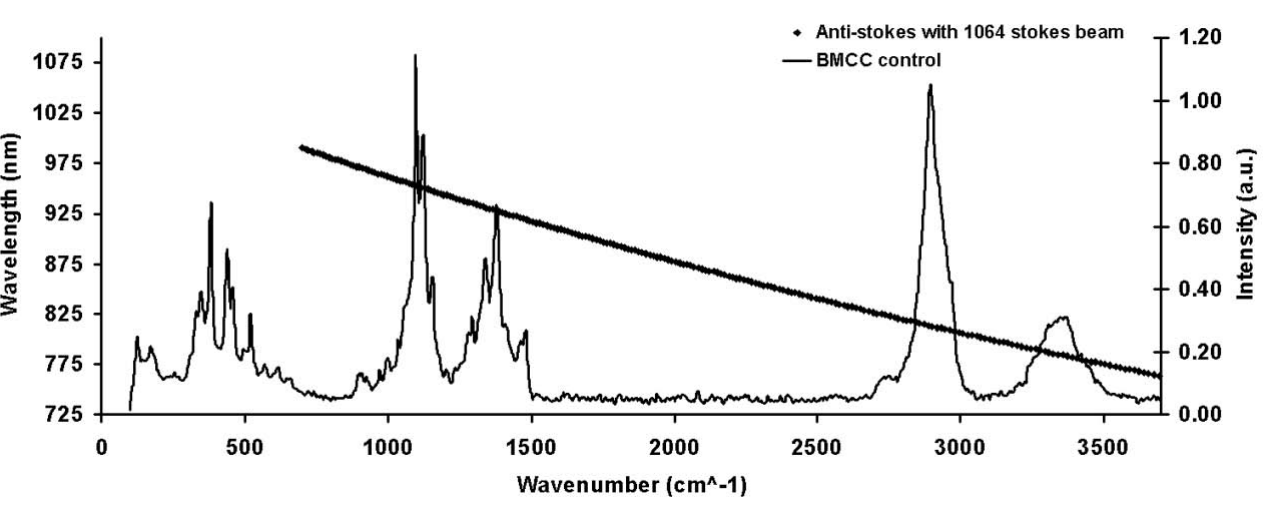
**Spectrum showing the comparison between the anti-Stokes signals generated from the Raman vibrational modes excited by the existing CARS system and the actual FT-Raman spectrum of bacterial microcrystalline cellulose (BMCC)**.

It appears that work to combine SPM with near-field optical spectroscopy is still in its infancy, considering that very few studies have been performed on complicated biological materials such as the plant cell wall. In order to take advantage of the recent developments in near-field spectroscopy and apertureless SPM for the characterization of plant biomass, we have designed a new system that combines a CARS microscope (Figure 3) and an AFM (Figure 4). In this system, a Neodymium-doped yttrium orthovanadate (Nd:YVO_4_) 1064 nm laser is frequency doubled (532 nm) to pump an optical parametric oscillator (OPO) that converts the pump laser line into two output waves of lower frequency, signal (ω_s_) and idler (ω_i_) by means of a non-linear lithium triborate (LBO) crystal. Using custom optical components, the two beams are collinearly added by means of a dichroic mirror and coupled to a component-built microscope. The output from this microscope is directed into both a spectrometer and a photomultiplier tube for CARS signal acquisition. Additional epi-detection is required to collect the near-field CARS signal that can be coupled into a photomultiplier tube with photon-counting capability, permitting quantitative measurements. AFM with metal-coated probes can be used to generate the TE-CARS effect. In this case, both the photon-counting system and the AFM will be coupled together to a set of timing electronics, allowing the spectral signal to be modulated at the tip frequency. The use of the dichroic mirror that collinearly adds the Stokes beam with the pump beam will give a broad, excitable tunable wave number (cm^-1^) range between 700 cm^-1 ^and 3700 cm^-1^. This broad, excitable tunable range is demonstrated in Figure 5, which compares the FT-Raman spectrum of bacterial microcrystalline cellulose with the tunable anti-Stokes range of the CARS system. We believe that the TE-CARS system described here can easily excite the active Raman modes within a cell wall; however, it remains to be seen if the spatial resolution needed to chemically characterize cell wall structures, such as microfibrils, can be afforded by TE-CARS. In addition, the recently emerging imaging technique, stimulated Raman scattering (SRS) microscopy, could be used to further improve the chemical resolution of cell walls. We believe that SRS could be achieved using the same instrumentation developed for CARS measurements (and described above). SRS offers higher sensitivity and reduced background imaging of biological samples compared with CARS microscopy [45].

## Conclusion

SPM has enhanced our ability to directly visualize the topography of plant cell wall samples at the sub-nanometer scale. These imaging techniques have led to precise measurement of the diameters of the microfibrils, and insight into the arrangement of the microfibrils in cell wall lamellae. It has been demonstrated that the chemical resolution can be significantly improved when SPM is used in conjunction with apertureless probe and near-field spectroscopy. There has been a limited amount of work reported using the apertureless near-field technique for characterizing plant cell wall materials; however, from this work we can speculate that developing the ability to simultaneously collect topological information from AFM and use it in combination with chemical information from linear or non-linear optics will provide the information required to deepen our understanding of the architecture of the plant cell wall.

These techniques, however, harbor inherent challenges that must be overcome. For example, the fundamental problems associated with the auxiliary gap-width-sensing mechanism used for AFM operation contribute to tip artifacts and misinterpretation of the data. These tip artifacts can also cause crosstalk within the optical signals, leading to a misleading optical image as well. Along with these tip artifacts, it has been shown that for apertureless techniques there are competing signals that diminish the desired signal, and thus further work is needed to overcome these unwanted effects. The next challenge in advanced imaging of the plant cell wall using SPM is the demonstration that TE-Raman, TE-CARS or TE-SRS can be used successfully to collect chemical information at the scale of the plant cell wall layers (approximately 50 to 100 nm) or even the microfibril itself (approximately 5 to 50 nm).

## Competing interests

The authors declare that they have no competing interests.

## Authors' contributions

JY wrote the bulk of the paper and came up with the idea to write a journal article on this subject matter. MH was involved in the primary discussion of this paper as well as provided guidance and input. He was also involved in editing the revisions for this paper. SYD supplied the images for figure 1 as well as provide input and helped write a couple of sections, primarily dealing with the cellulose and was involved in editing the revisions for this paper.
